# A New Device for Thoracoscopic Cryoanalgesia in Pectus Excavatum Repair: Preliminary Single Center Experience

**DOI:** 10.3389/fped.2020.614097

**Published:** 2021-01-18

**Authors:** Michele Torre, Leila Mameli, Rachele Bonfiglio, Vittorio Guerriero, Lucia Derosas, Loredana Palomba, Nicola Disma

**Affiliations:** ^1^Pediatric Thoracic and Airway Surgery Unit, IRCCS Giannina Gaslini, Genoa, Italy; ^2^Pediatric Surgery Department, IRCCS Giannina Gaslini, Genoa, Italy; ^3^Anesthesiology and Pain Management Department, IRCCS Giannina Gaslini, Genoa, Italy

**Keywords:** MIRPE, pectus excavatum, cryoanalgesia, post-operative pain, thoracic surgery

## Abstract

**Introduction:** Cryoanalgesia has been recently described as alternative technique for immediate and persistent pain treatment after pectus excavatum repair. Cryoanalgesia has the potentiality to reduce analgesic consumption and length of hospitalization. However, cryoanalgesia has not been standardized yet: the previous reports describe different techniques and systems and include only small series. In Europe, no reports on cryoanalgesia for pectus repair have been published so far.

**Materials and Methods:** This is a prospective single center pilot study performed in adolescents undergoing minimally invasive pectus excavatum repair with a new cryoanalgesia system, using a probe designed specifically for thoracoscopy. This new double lumen probe has the theoretical advantage of freezing only in its tip, so reducing the risk of complications.

**Results:** Seven patients undergoing pectus excavatum repair were treated with cryoanalgesia performed with the new probe. No complications of cryoanalgesia were reported. Total consumption of morphine during hospital stay was between 0.1 and 0.35 mg/kg, with no side effects reported. Mean time to discharge was 2.4 days. All patients reported a good pain control with a fair need of rescue medications for pain relief during the first week after discharge, and a very good pain control without need of rescue medications during following weeks.

**Conclusions:** Our pilot study showed that the new cryoanalgesia device is efficacious in terms of pain control, hospital stay and resumption of post-operative activities. The cryoprobe designed allowed an easy and safe maneuver. A prospective trial is needed to better define the risks and benefits of this technique.

## Introduction

Mini-invasive repair of PE (MIRPE) has gradually become the preferred surgical approach to correct pectus excavatum (PE), the most common chest wall anomaly (1). During MIRPE, one or more retrosternal bars are placed substernally to lift the sternum up; the bars will be removed after approximately 3 years. Physical exercises and physiotherapy after MIRPE are an important part of the post-operative rehabilitation program. One of the drawbacks of PE repair is the severe post-operative pain, which can persist after discharge and delay the return to normal activities (school, sport, etc.), despite pharmacological treatment (2). Early post-operative pain is commonly managed with continuous epidural infusion (or intravenous opioids or a combination of these) for 3–4 days, the long lasting pain is treated with NSAIDs and minor opioids (3).

Cryoanalgesia has been recently described with promising results as alternative technique for immediate and persistent pain treatment after PE repair (4–6). Cryoanalgesia disrupts the nerve structure and creates wallerian degeneration, but leaves the myelin sheath and endoneurium intact (7).

According to the recent literature on cryoanalgesia in MIRPE, published from North and South America, cryoanalgesia has the potentiality to reduce analgesic consumption and length of hospitalization, but also to ameliorate persistent pain and facilitate return to normal activity (8–13). However, the technique of cryoanalgesia was not homogeneous: in the reports available so far, different protocols, probes and different cryogenics systems were used, manufactured by US Companies (©AtriCure, Inc., Mason OH; Frigitronics®, Connecticut). Technically, the same principle -cryoanalgesia- can be delivered using different machines and probes, both percutaneously or thoracoscopically, and using various protocols. In Europe, Metrum Cryoflex (Warsaw, Poland, EU) has recently designed a special probe specifically for intrathoracic use during MIRPE, consisting of a hollow cannula containing an inner smaller cannula (“double lumen”). This represents an advantage with respect to the other probes: an ice ball is generated just at the tip of the cryoprobe, allowing a safe and precise treatment. To our knowledge, no experience about trans-thoracic cryoanalgesia for MIRPE was reported in Europe so far.

Cryoanalgesia has been introduced in our hospital, one of the referral center for MIRPE repair in our Country, with an average of 60 patients per year operated. However, before starting a proper trial it was decided to run a pilot to determine feasibility and efficacy of this approach, performed with the new probe, as well as potential limitations and side effects. Then, the aim of this article is to describe our preliminary experience and provide an input for future research on this field.

## Materials and Methods

This is a prospective single center pilot study performed between December 2019 to January 2020 in adolescents undergoing MIRPE with cryoanalgesia treatment.

### Ethics

The study involving human participants was reviewed and approved by IRB (authorization n.31435/20). The participants (legal guardians) provided written informed consent to participate in this study.

### Anesthesia Technique

Anesthesia management consisted of total intravenous anesthesia with propofol and remifentanil titrated in order to obtain a bispectral index (BIS) range of 40–60 and muscle relaxation with rocuronium. A double lumen tracheal tube was inserted for lung one lung ventilation. When thoracoscopy was installed, the ventilation was temporarily suspended on the ipsilateral lung of the side for cryoanalgesia application. At the end of the cryoanalgesia, bilateral lung ventilation was started, and the MIRPE was performed.

Post-operative analgesic protocol was based on PCA of morphine with no background infusion and ketorolac plus acetaminophen as rescue analgesia, plus oral benzodiazepine for muscle relaxation.

### Cryoanalgesia Technique

**Figure 1:**
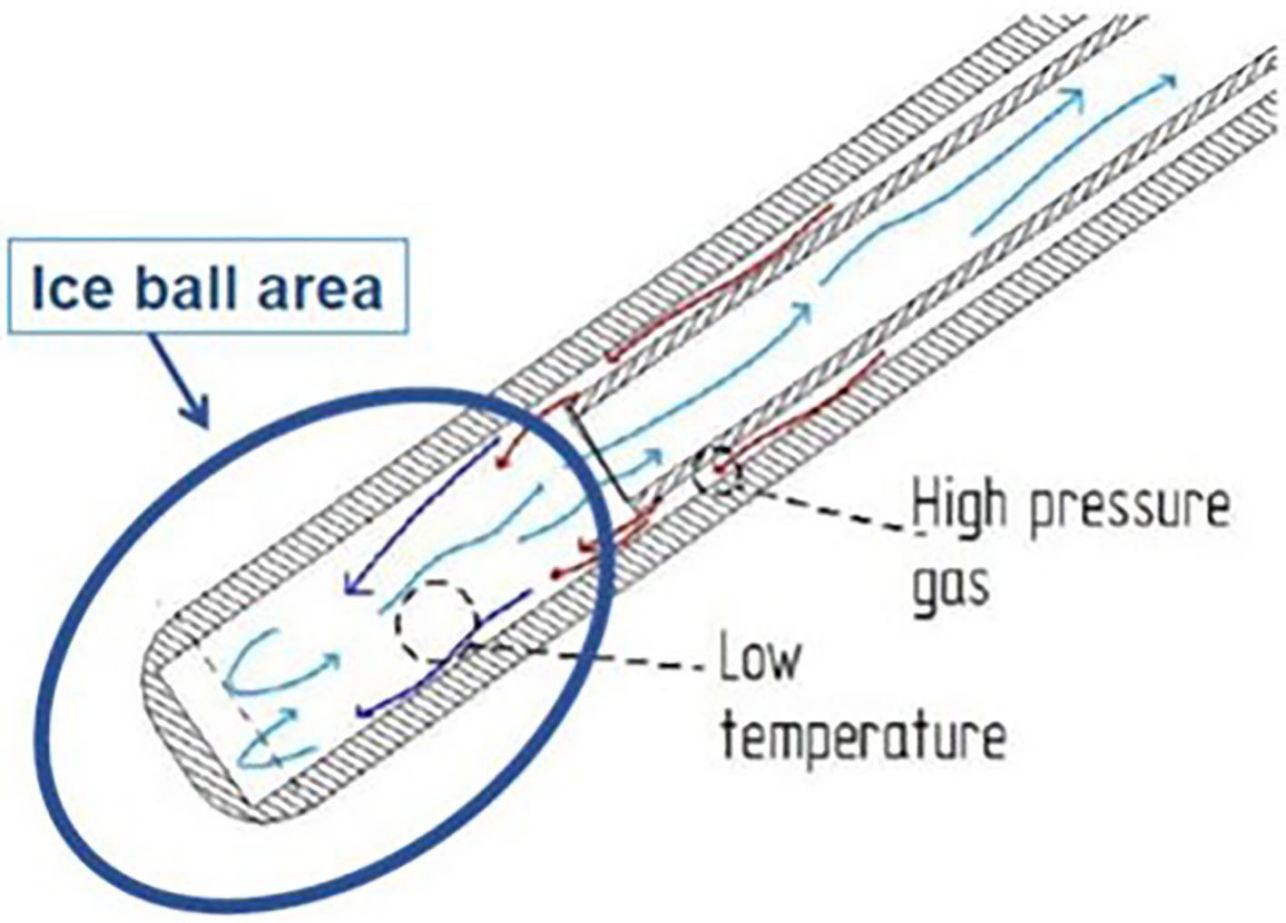
The cryoanalgesia probe with the double cannula, which allows the creation of the ice ball just at the tip of the probe.

**Figure 2:**
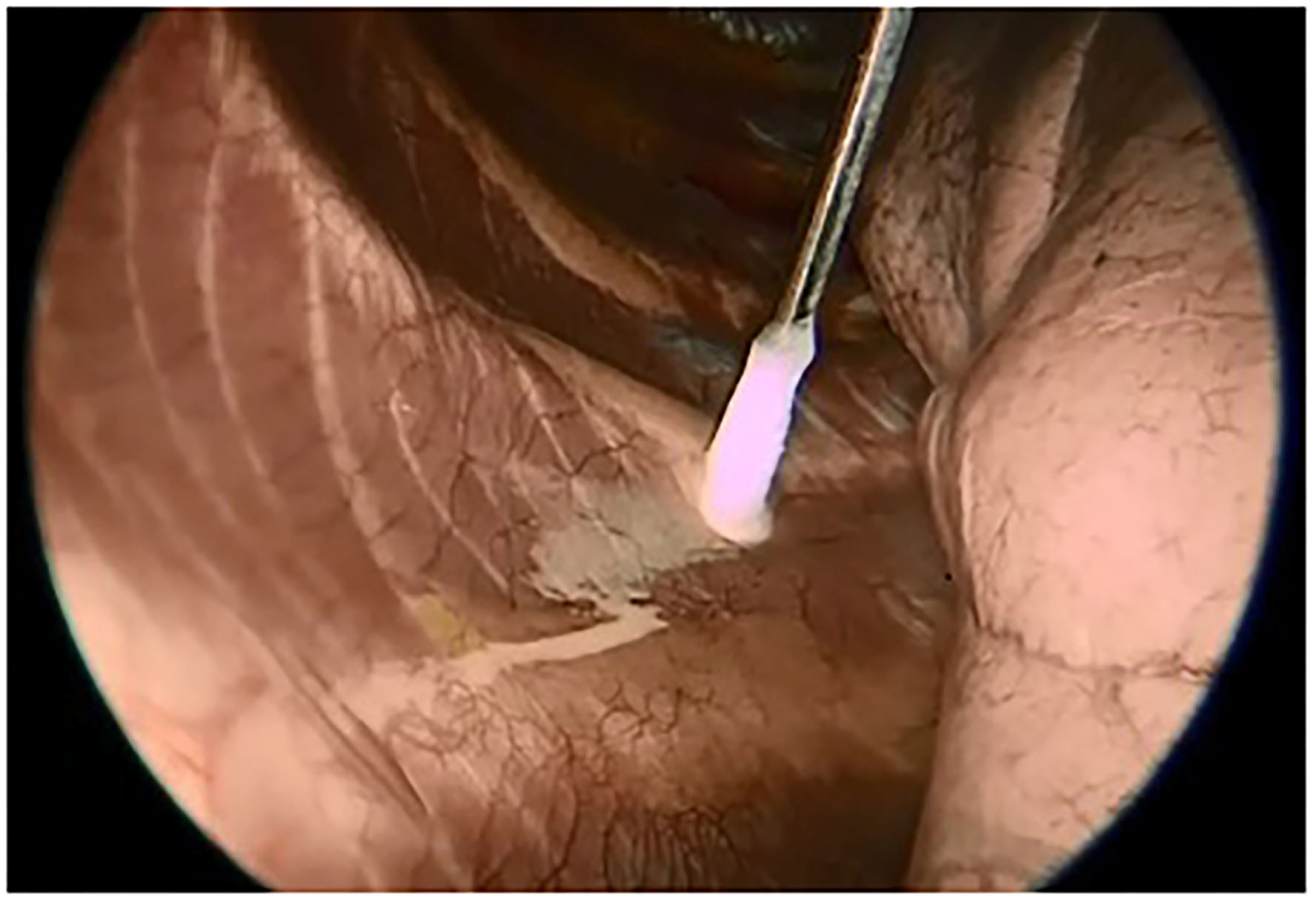
Thoracoscopic view of the ice ball.

A specifically designed cryoanalgesia probe for thoracoscopic use was adopted in this case series (Figure 1). This cryoanalgesia system was designed to exploit the physical performances of carbon dioxide (CO_2_ at −70°C), without reaching CO_2_ boiling point (−79°C), hence ensuring the reversibility of the nerve injury and the lack of effect on the connective structures. The dedicated cryoprobes were specifically designed with a “double lumen,” differently from the “single lumen” probes, in which the gas flows toward the whole length of the probe, generating the freezing effect on the whole probe length. Cryo-S Painless device and double lumen cryoprobe manufactured by a Polish company Metrum Cryoflex consists of two cannulas. One of them is located along the external wall of the probe and the other one goes through the center of the probe for the gas to flow. Pressurized CO_2_ flowing between two cannulas provides probe dynamic isolation. The rapid expansion of the gas can only be observed at the tip of the probe (Joule–Thompson effect). As a result of the temperature drop, an ice ball is generated just at the tip of the cryoprobe, allowing a safe and precise treatment. CO_2_ is then vented through the inner cannula, no gas is released into the body. For the thoracoscopic use, a 12 G, 35 cm long blunt tip atraumatic probe was produced. The purpose was to treat 5 nerves (from T3 and T8) on each side in every patient undergoing MIRPE with cryoanalgesia. The probe was inserted on the same side of thoracoscopy (first on the right side, than on the left side) and kept in contact with the posterior aspect of the selected intercostal nerve for 2 min at −70°, under direct visual control. A small ice ball formation indicated that the probe was functioning properly (Figure 2).

### Intra- and Post-operative Evaluations

The following intraoperative data were collected: total surgical time, number of nerves treated, opioid consumption, time to awake from anesthesia and respiratory dynamic at awakening. Post-operative evaluations included sleep quality, pain at rest and during coughing, breakthrough pain, total morphine consumption, any side effect, and duration of hospitalization. Data were collected every 4 h until discharge through a self-assessment questionnaire. Level of anesthesia was evaluated by cold test once at discharge. The patients were then followed for 3 months after discharge: 1 month after surgery, when we evaluated patients in clinic for a physical examination and a self-assessment questionnaire was given (Cali 9) to evaluate ability in daily routine and activity limitations (14). Three months after surgery, patients were interviewed during a telephone call, and the following data were collected: pain at rest, breakthrough pain, sleep quality, extension of analgesia, and/or paresthesia, need of rescue pain medications. Any adverse event was reported at any time.

## Results

This pilot study included seven patients undergoing MIRPE. Demographic data are summarized in Table 1.

**Table 1:** Demographic data.

**Patients**	**Sex**	**Age (years)**	**Weight (Kg)**	**Length (cm)**	**Haller index**
1	M	17	56	176	5.9
2	M	18	80	189	3.2
3	F	20	55	168	10.4
4	M	16	48	169	11.6
5	M	15	55	173	N.A.
6	M	17	63	179	4
7	M	16	60	185	4.7

### Surgical Results

**Figure 3:**
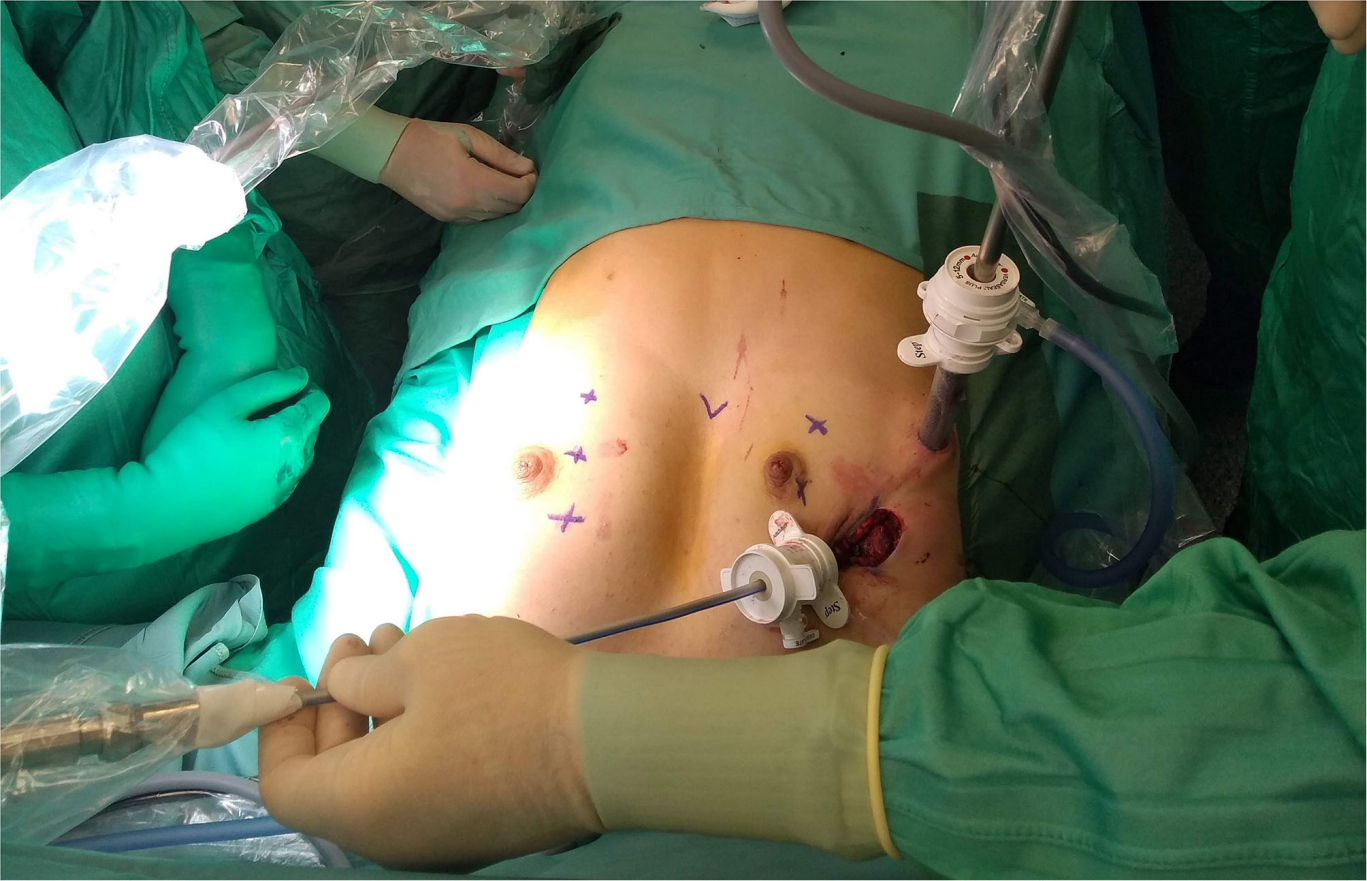
Probe insertion through a 5 mm. Trocar placed at the site of surgical incision.

**Figure 4:**
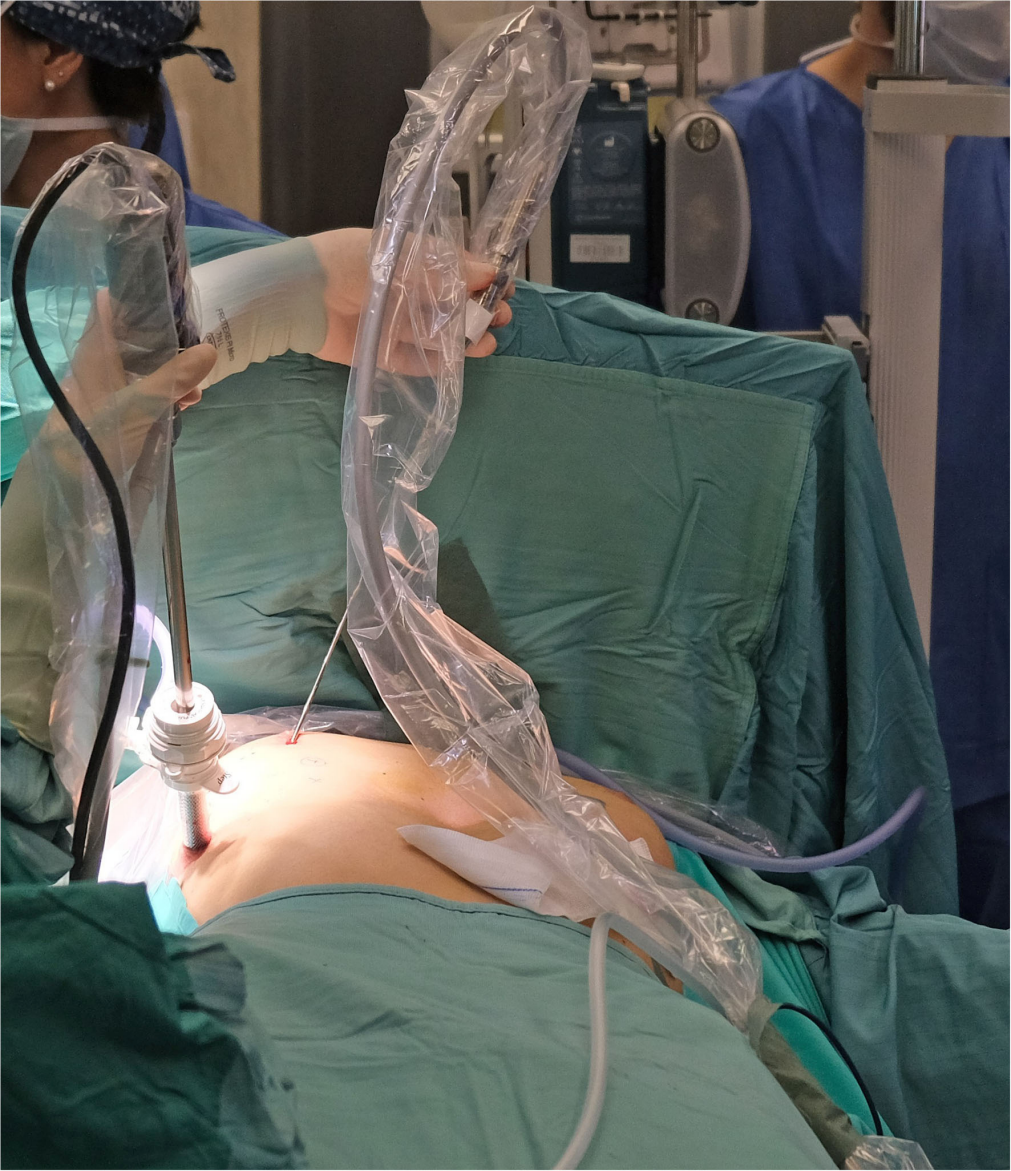
Trocarless percutaneous probe insertion in a medial position.

In all patients cryoanalgesia was successfully performed before standard MIRPE. After the first 4 patients, it was decided with the manufacturer to additionally improve the cryo-performance, based on challenging situation generated by the core heat counteracting the generation of the freezing effect. The cryoprobe microchip was calibrated to obtain a gas flow able to adequately perform. The dedicated cryoprobe was designed to generate a 3 cm long ice ball providing the desired freezing effect at the very end of the probe only. The equipment allowed performing the procedure in both automatic or manual mode. Surgical approach was also slightly modified. In the first 4 patients, the probe was inserted into the thorax through a 5 mm port, placed at the level of skin incision (Figure 3). In the last three patients, it was preferred to insert the probe through a further more medial percutaneous trocarless access, in order to have a better access to the posterior aspect of the ribs and the intercostal nerves (Figure 4).

### Pain Evaluation

Table 2 shows intra-operative details and post-operative immediate results. No complications were observed. Average surgical time of cryoanalgesia and MIRPE was 140 min. Cryoanalgesia time lasted 50–70 min. The number of intercostal nerves treated ranged from 4 to 6 each side (most commonly 5 each side).

**Table 2:** Intraoperative data.

**Patients**	**Total surgical time (min.)**	**Right: number of nerves treated (levels)**	**Left: number of nerves treated (levels)**	***N.^**°**^* of bars**	**Intraoperative opioid consumption (mcg)**	**Respiratory dynamic at awakening**	**Time to awareness (min.)**
1	100	5 (3°-7°)	6 (2°-7°)	2	1,800	Good	30
2	150	5 (3°-7°)	5 (3°-7°)	3	5,500	Good	40
3	210	6 (2°-7°)	6 (2°-7°)	2	2,000	Good	15
4	160	5 (3°-7°)	5 (3°-7°)	3	4,000	Good	30
5	135	5 (T3–T7)	4 (T3–T6)	2	2,520	Good	20
6	90	6 (T3–T8)	6 (T3–T8)	2	1,840	Good	10
7	135	5 (T3–T7)	6 (T3–T8)	1	2,000	Good	10

Table 3 shows the results of the in-hospital analgesia evaluation. The sleep quality was good in all patients. Total consumption of morphine during hospital stay was between 0.1 and 0.35 mg/kg, with no side effects reported. Mean time to discharge was 2.4 days.

**Table 3:** Post-operative data.

**Patients**	**Sleep quality**	**Pain at rest**	**Breakthrough pain**	**Total morphine Iv (mg)**	**Morphine iv per kg (mg/kg)**	**Level of anesthesia (right side)**	**Level of anesthesia (left side)**	**Side effects**	**Post-op. day of discharge**
1	Good	3	4	6	0.1	T3–T5	T3–T6	No	2
2	Good	3	4	8	0.1	T3–T6	T5–T8	No	2
3	Good	2	2	13	0.2	T2–T6	T2–T7	No	2
4	Good	3	4	11	0.2	T3–T6	T2–T6	No	2
5	Good	3	4	19	0.3	T3–T10	T3–T7	No	4
6	Good	3	4	10	0.15	T3–T10	T4–T10	No	3
7	Good	3	4	21	0.35	T4–T8	T4–T9	No	2

*Pain at rest and breakthrough pain were assessed with NRS (numerical rating scale, with a score from (0 to 10)*.

### Follow Up

All patients reported a good pain control with a fair need of rescue medications for pain relief during the first week after discharge, and a very good pain control without need of rescue medications during following weeks.

All patients came back to school and social activity within the first month after discharge and they all referred problems with the fast walking only during the first week.

## Discussion

Results from this initial experience confirm cryoanalgesia is a promising technique for post-operative analgesia after MIRPE. The technique resulted in good post-operative analgesia and, as consequence, in reduced use of analgesics and early return to normal life. Comparing the patients treated with cryoanalgesia and the standard PE patients in our Institution, the first were discharged earlier [2.4 days with respect to 6 days of the standard group (15)].

However, despite no relevant side effects, a well-performed prospective trial is needed to understand risks and benefits of this technique.

Considering that time required for MIRPE was 82 min in our overall series of 600 patients (15), MIRPE operative time was increased significantly for cryoanalgesia. The surgeon and patients have to be aware of this before the surgery. However, we have to consider that this time will be probably reduced when the procedure will be performed routinely, and that in these patients the time for peridural catheter positioning is saved.

Cryoanalgesia creates wallerian degeneration without interrupting the myelin sheath and endoneurium, so we prefer the term of “cryoanalgesia” to the term “cryoablation” often reported in the literature and suggesting the (wrong) idea of removing something. Cryoanalgesia proved to be useful in terms of reduction of opioid consumption and hospital stay in previous experiences in North and South America [US (9–12) and Argentina (13)]. Our experience is consistent with the other reported experiences, even if the protocols adopted and the cryogenic systems used are different from place to place. This makes comparison difficult.

In our experience, we have been using CO_2_ as cryogenic gas, differently from previous reports (9–12), reporting the use of N_2_O. CO_2_ and N_2_O have similar melting points (−79 and −89°C, respectively), while the temperature needed for axonotmesis is between −15 and −35°C. Our data seem to demonstrate that CO_2_ is effective as well as N_2_O in order to achieve the result, but it could be interesting to compare in future the effects of the two gases.

Parrado et al. (16) compared post-operative consumption of morphine in three different groups (cryoanalgesia, epidural, and multimodal analgesia) and they found that opioid consumption was lower in cryoanalgesia group (237 mg). In our pilot study the average value of total opioid consumption was 7.5 mg in line with results of Morikawa et al. (17). Our patients showed a length of stay of 2.4 days, in line with other studies [1.9 days in Grave et al.'s (11), 2.2 ± 0.4 in Morikawa et al.'s (17)].

In our experience, we did not find a 24 h (or more) delay of the peak cryoanalgesic effect, as reported by other Authors (18). Graves et al. (11) described a gradual sensitivity recovery with a latero-medial progression, patients showed a total sensitivity recovery in 4 months. In our pilot study patients reported no more analgesia after 2 months.

Another point to be clarified is the preferred approach for the cryonalagesia probe insertion. The first described was the transmediastinal, with the sternum elevated, mediastinal dissection performed, and the probe passed substernally toward the contralateral side (4). The transmediastinal approach was chosen to reach the intercostal nerve perpendicularly and to overcome the difficulty of the ipsilateral approach due to the curvature of the ribs. Later, ipsilateral approach gained popularity when some technical tricks were introduced to make it possible to flex the malleable probe while providing insulation (16). We have adopted the ipsilateral approach without the need of insulating the probe (for the features explained above). Inserting the probe trocarless more medially than the MIRPE incisions allowed to reach all the posterior aspect of intercostal spaces in a safe and easy way.

In Europe, no reports of cryoanalgesia in MIRPE have been published yet. Our aim was to test MetrumCryoflex system and its efficacy on pain control, using special thoracoscopic probes created specifically for MIRPE cryoanalgesia. Some important technical details specific of these probes are worthy to be underlined. First, the length and the strength of the probe allowed a safe, precise, and easy surgical approach. The equipment can be used in both manual/automatic mode according to the clinicians need. In all cases we were able to reach 5 or 6 intercostal levels. The dimensions of the probe (length and diameter) were optimized for a thoracoscopic use, creating an ergonomic tool allowing a percutaneous approach and an easy approach to the selected nerves. In our experience, the improved probe used in the last three patients was more ergonomic than the first and the procedure resulted easier to perform. Moreover, the double lumen cannula allowed ice ball formation exclusively at the very end of the probe tip, with the probe sheath not freezing. This technical detail reduced the risks of complications related to tissue cold injury that are seldom reported with other types of cryoprobes (in particular pneumothorax and skin lesions) (15) and allowed us to insert the cryoprobe percutaneously, without using a trocar.

The cryoprobe is atraumatic (blunt tip) and echogenic; the ice ball could be detectable also with ultrasound imaging if percutaneous approach will be preferred. Percutaneous cryoanalgesia ultrasound guided with paravertebral nerve blockade could be an attractive alternative in PE patients. We do not have experience of this alternative approach and in our opinion comparative studies between thoracoscopic and percutaneous approach are warranted.

In conclusion, our pilot study showed that cryoanalgesia is efficacious in terms of pain control, hospital stay and resumption of post-operative activities. The cryoprobe designed allowed an easy and safe maneuver.

We acknowledge there are many limitations with this preliminary results, mainly due to the small sample of patients and the heterogeneity of the technical details. However, our result confirms that cryoanalgesia is a promising tool to treat post-operative pain of MIRPE. A large prospective study with a strict follow-up will definitely clarify is cryoanalgesia is the technique of choice for MIRPE.

## Data Availability Statement

The raw data supporting the conclusions of this article will be made available by the authors, without undue reservation.

## Ethics Statement

The studies involving human participants were reviewed and approved by IRB Gaslini Institute. Written informed consent to participate in this study was provided by the participants' legal guardian/next of kin.

## Author Contributions

MT designed the study, performed the procedures, and wrote the main part of the paper. LM participated to the design of the study, participated to the procedures, and evaluated the pain level in the post-operative periods. RB participated to the procedures, evaluated the pain level during the post-operative and follow-up periods, and contributed to the drawing of the paper. LP and LD evaluated the pain level during the post-operative and follow-up periods and analyzed the data. ND participated to the design of the technique and reviewed the manuscript. All authors contributed to the article and approved the submitted version.

## Conflict of Interest

The authors declare that the research was conducted in the absence of any commercial or financial relationships that could be construed as a potential conflict of interest.
